# Impact of different controlled temperature and humidity conditions on the behaviour, posture and spatial needs in fattening rabbits

**DOI:** 10.3389/fvets.2025.1653718

**Published:** 2025-08-07

**Authors:** Alexandra Contreras-Jodar, Antoni Dalmau, Marc Bagaria, Marilys Rendon, Ahmed A. K. Salama, Antonio Velarde

**Affiliations:** ^1^Institut de Recerca i Tecnologia Agroalimentàries (IRTA), Animal Welfare, Girona, Spain; ^2^Department of Agri-Food Engineering and Biotechnology (DEAB), Escola d'Enginyeria Agroalimentària i de Biosistemes de Barcelona (EEABB), Universitat Politècnica de Catalunya, BarcelonaTech (UPC), Barcelona, Spain; ^3^Ruminant Research Group (G2R), Department of Animal and Food Sciences, Facultat de Veterinària, Universitat Autònoma de Barcelona, Barcelona, Spain

**Keywords:** rabbit, transport, space allowance, container height, posture, behaviour, thermal conditions, welfare

## Abstract

This study aimed to evaluate the effect of thermal conditions on the posture and behaviour of fattening rabbits and to determine the minimum space allowance and height required in a transport container to allow for thermoregulatory behaviours. Thirty-six rabbits (2.27 ± 0.13 kg) of hybrid genetics were fasted for 6 h and then exposed to three thermal treatments (T1: 15.3°C and 63.0% RH; T2: 22.9°C and 38.3% RH; T3: 31.1°C and 35.5% RH) for 8 h in a climate-controlled chamber. Postural and behavioural responses were continuously recorded via video and subsequently analysed using standardised ethograms. These responses were also analyzed by comparing the first and last 4 h of exposure to assess temporal changes in response to thermal treatments but no significant differences were found between them. The space occupied by each rabbit was quantified through digital image analysis. Results showed that the warmer the thermal treatment, the more frequently the rabbits adopted sprawled postures, while self-grooming and exploratory behaviours decreased and inactivity increased significantly. Rabbits tend to avoid physical contact under higher temperatures, likely as a mechanism to enhance heat dissipation. The space occupied varied with posture: 168 cm^2^/kg when lying, 205 cm^2^/kg in sternal sprawl, and 277 cm^2^/kg in lateral sprawl, corresponding to a stocking density of 60, 49, and 36 kg/m^2^, respectively. Height needed to perform the different behaviours ranged from 13 cm in sprawled positions to 32 cm when upright. During the 8 h treatment, rabbits spent approximately 1% of the time in upright posture, suggesting limited use of vertical space. In T1, rabbits require a minimum of 168 cm^2^/kg (60 kg/m^2^) to lie down all at the same time. In T2 and T3, rabbits require 205–277 cm^2^/kg (36–49 kg/m^2^) to allow sprawling in static, non-ventilated conditions. A cage height of ≥ 35 cm is necessary for rabbits of this weight to stand upright. These findings highlight the importance of adjusting space allowance and height in transport containers based on thermal conditions to safeguard rabbit welfare during transport. These experimental results need validation under commercial transport conditions.

## Introduction

Approximately 74 million rabbits were transported from farms to slaughterhouses in the European Union (EU) in 2024 (European Rabbit Association (ERA), personal communication). The transport of rabbits within the same Member State for slaughter accounts for the majority of journeys, and 99% of transport is carried out by road (1). Rabbits are usually fasted for 4–6 h prior to be transported (ERA, personal communication) and the journey time is usually under 4 h in all EU countries, with some exceptions (e.g., rerouting to another slaughterhouse due to flooding), where it can be up to 8 h (ERA, personal communication).

The legal framework for the protection of animals during transport is provided by the Council Regulation (EC) No 1/2005 of 22 December 2004 (2). However, there is a lack of specific requirements for rabbits in the current EU legislation. As a result, the stocking densities used in practice depend largely on the know-how of each integrator (3, 16).

In 2020, the “Farm to Fork” Strategy was launched with the aim of reviewing and updating EU legislation on animal welfare. Consequently, the European Commission requested the European Food Safety Authority (EFSA) to conduct a scientific review of current animal transport practices from an animal welfare perspective. As a result, in September 2022, EFSA published five scientific opinions, one of which focused on poultry and rabbits (1).

As a guideline, EFSA (1) suggested that the space allowance for fattening rabbits with a commercial body weight of 2.0–2.5 kg corresponds to the area occupied by a rabbit lying in a resting posture calculated using the allometric formula proposed by Petherick and Phillips (4). Therefore, this results in 200–215 cm^2^/kg (equivalent to a stocking density of 47–50 kg/m^2^). This is slightly higher than the area a rabbit occupies in lying position, which has been quantified at 176–194 cm^2^/kg or 52–57 kg/m^2^ later by Giersberg et al. (5) through planimetric measurements. Additionally, EFSA (1) reported that the height of the containers should allow rabbits to sit in a natural upright posture without their ears touching the ceiling of the container which was suggested to be 35 cm for rabbits up to 3 kg. Otherwise, they may experience movement restriction and be unable to stretch their ears, which would impair their ability to dissipate heat under thermally challenging conditions. However, the EFSA report (1) also acknowledges significant knowledge gaps. In particular, it remains unclear whether rabbits would actually adopt this upright sitting posture on their hind legs during transport, even if sufficient height were available. More broadly, there is a lack of scientific data on how thermal conditions during transport affect rabbit posture and behaviour. Although rabbits are known to be highly sensitive to heat stress, no studies to date have systematically assessed how different temperature and humidity levels influence their behavioural responses, such as postural adjustments for thermoregulation, in the confined conditions of commercial transport crates. This lack of empirical evidence limits the ability to validate or refine current recommendations on space and container design for transported rabbits.

Following the EFSA report (1), in December 2023, the European Commission presented a proposal to revise Council Regulation (EC) No. 1/2005, with the aim of improving animal welfare during transport and aligning the legislation with best practices in animal welfare management. However, rabbits remain one of the least-studied species in this context, and scientific evidence on the necessary space and appropriate container height and the posture and behaviour in the transport crates in relation to ambient temperature and humidity during transport, remains limited.

Therefore, the objective of this study was to simulate in a climate chamber, the effect of different thermal conditions (combinations of temperature and humidity) during road transport (4 and 8 h to represent the most common and the maximum transport durations in the EU) in rabbits previously submitted to feed and water deprivation for 6 h (commercial fasting) on the posture and behaviour of fattening rabbits. These findings will support the evaluation of the minimum space allowance and height that enables rabbits to perform effective its full range of thermoregulatory behaviours in a transport container.

## Materials and methods

The animal care conditions and handling practices of the study were approved by the Ethics Committee for Animal and Human Experimentation (CEEAH) of the Universitat Autònoma de Barcelona (UAB) under protocol CEEAH 5682-CEEA-UAB on April 23, 2024.

### Animals, housing and treatments

Thirty-six healthy fattening rabbits (males and females) of hybrid genetics, with no lameness and a homogeneous body weight (2.272 ± 0.126 kg live weight), were transported from a commercial farm to the Servei de Granges i Camps Experimentals (SGCE) of the UAB in Bellaterra (Barcelona, Spain). The rabbits were housed in groups for 3 days in pens with slatted floors and given access to feed (the same diet as on the farm of origin) and water *ad libitum* to recover from the stress caused by transport to the SGCE.

Truck transport environmental conditions were simulated in a climate chamber measuring 4 m × 6 m × 2.3 m, equipped with a temperature and humidity control system (Carel Controls Ibérica, Barcelona, ES). The rabbits were divided into three groups balanced by weight and randomly assigned to one of three environmental treatments (20, 25, and 30°C at 50% relative humidity). However, the climatic chamber provided temperature and relative humidity values different from those programmed due to a previously undetected malfunction in the humidification system leading to the following actual thermal treatments: T1 (15.3°C and 62.9% relative humidity), T2 (22.9°C and 38.3% relative humidity) and T3 (31.1°C and 35.5% relative humidity) for 8 h.

Prior to the thermal treatment, rabbits underwent a 6-h fast from feed and water to simulate commercial fasting conditions. They were then weighed (WA200, MeierBrakenberg, Brakenberg, DE) and moved to the climate chamber (located adjacent to the housing pens). The chamber was equipped with three cages measuring 105 cm × 62 cm × 35 cm (Penta Cage 210,950, Copele, El Palmar, ES). Each cage had an internal wire mesh divider, allowing it to be split into two equal compartments.

Two rabbits were allocated in each of the six compartments, resulting in a total of 12 rabbits per thermal treatment. The available surface area was 3,255 cm^2^ (i.e., 714 cm^2^/kg of rabbit).

The chamber was also equipped with three video cameras (IP Camera DH-IPC-HDW2231TP-ZS-S2, Zhejiang Dahua Vision Technology Co., Ltd., Hangzhou, CN) connected to a digital image recorder (network video recorder DHI-NVR4108-8P-4KS2/L, Zhejiang Dahua Vision Technology Co., Ltd., Hangzhou, CN). Each cage had one camera assigned, positioned in a nadir perspective to allow continuous monitoring of rabbit behaviour. One temperature and relative humidity sensor (HOBO MX2301 Temp/RH Data Logger, HOBO Data Loggers, Bourne, Massachusetts, US) was placed in each cage at rabbit head height and set to automatically record data every minute. Each thermal treatment lasted 8 h, during which temperature and humidity conditions were controlled.

**Figure 1:**
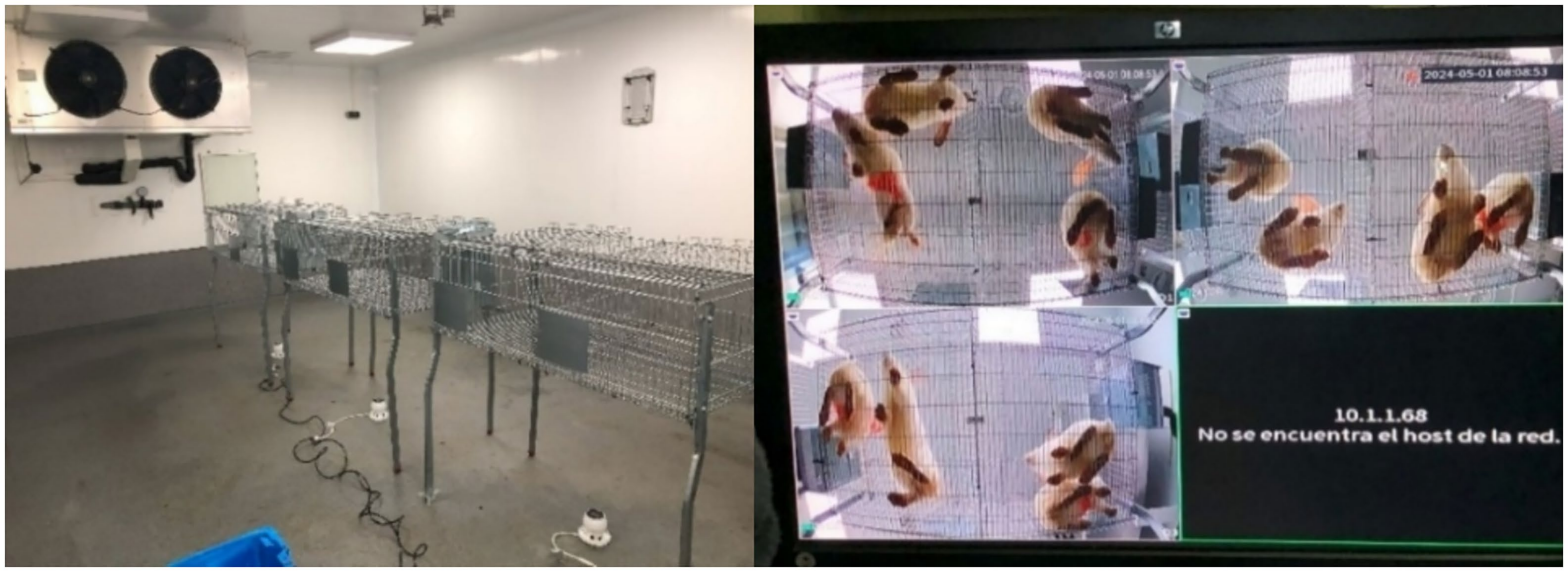
Layout of the cages, video cameras, and temperature and humidity sensors inside the climate chamber (left), and example of a nadir-perspective video recording showing the rabbits.

The layout of the cages, video cameras, and temperature and humidity sensors inside the climate chamber, along with an example of the video recording, is shown in Figure 1.

### Space and height occupied

Images were extracted from the 8-h video recordings by capturing one frame every 5 min. The images were processed using DaVinci Resolve 19 software to correct the visual distortion characteristic of surveillance cameras (i.e., fisheye lenses). Once the distortion was corrected, the pixels corresponding to the area occupied by each rabbit were quantified using MIP45 (Digital Image System S. L., ES).

### Posture and behaviour

The posture and behaviour of each rabbit were recorded at 5-min intervals using BORIS (Behavioural Observation Research Interactive Software) v.7.13.8 (6), following the ethogram presented in Table 1. Additionally, for each observation interval, it was noted whether the rabbits in each cage were in physical contact with their cage mates or not.

**Table 1:** Ethogram of the posture and behaviour of rabbits exposed to different combinations of temperature and humidity.

Category	Item	Definition
Posture	Sitting	Supported on the hind legs in a posture similar to that of a sitting dog, or with all four limbs extended and in contact with the floor.
	Upright	Standing with both hind legs firmly on the ground and the front legs lifted off the ground.
	Lying	In sternal recumbency, with all four limbs bent and resting on the ground, and the head held upright in an alert position.
	Sternal sprawled	Lying with the entire sternal region in contact with the cage floor.
	Lateral sprawled	Lying with a rotation of the lower trunk causing the hind legs, especially the thighs, to rest on the cage floor.
Behaviour	Walking	Moves a distance by changing the position of the hind legs.
	Hopping	Runs or jumps quickly and spontaneously, moving the hind legs side to side. This behaviour may involve abrupt movements and turns, often with short hops or dashes within the cage.
	Exploring	Contact of the tongue or nose with parts of the cage or a cage mate, related to sniffing behaviour.
	Self-grooming	Rabbit’s mouth or nose in contact with its own body, associated with grooming behaviours (15).
	Allogrooming	Rabbit’s mouth or nose in contact with another rabbit’s body, associated with grooming behaviours, absence of conflict, and establishment of trust within the group (15).
	Social negative	Interactions between individuals involving physical aggression.
	Inactivity	Immobile. No motor activity or any of the behaviours described above.

### Statistical analysis

Data on body weight, ambient temperature and relative humidity, behaviour, occupied space, and height were pre-processed, statistically analysed, and graphically represented using R software v.4.3.2 (7). Statistical significance was set at *p* < 0.05 for all analyses.

Body weight was analysed using analysis of variance (ANOVA) to compare the mean between treatments.

The analysis of posture and behaviour included the percentage of events in which each rabbit performed the postures or behaviours described in the ethogram, according to the thermal treatment. Four observers independently scored rabbit behaviour using the ethogram described in Table 1. Inter-observer reliability was assessed using Fleiss’ kappa coefficient (*κ*), which measures agreement among multiple raters. For this analysis, behavioural evaluations from 12 h of video footage were used, evenly distributed across the three thermal treatments. The resulting κ was 0.893, indicating excellent repeatability according to the classification by Fleiss (8). Therefore, the behavioural assessments were considered reliable for further analysis.

Data were initially analysed using a linear model that included the fixed effects of thermal treatment (T1, T2, and T3), period (P1: sum of events during the first 4 h; P2: sum of events between hours 4 and 8), and the interaction between thermal treatment and period. If the set of postures or behaviours did not vary over time or if there was no significant interaction between thermal treatment and period, these variables were removed from the initial model.

The available space per rabbit was calculated by dividing the occupied area (cm^2^) by its body weight (kg) and the mean was compared between treatments using ANOVA, with multiple comparisons corrected using the Tukey HSD method.

## Results

The rabbits had a similar initial body weight after fasting across treatments (2.272 kg ± 0.124; *p* = 0.278). The measurements obtained from the temperature and relative humidity sensors in each of the cages placed in the climate chamber are summarised in Table 2. As shown, there were no statistically significant differences between cages, indicating that all rabbits were exposed to similar temperature and relative humidity conditions during the experimental treatment (*p* > 0.05).

**Table 2:** Mean (±SD) ambient temperature and relative humidity according to the thermal treatment applied in the climate chamber.

Thermal treatment	Temperature, °C	Relative humidity, %	*p*-value
T1	15.3 ± 0.8	62.9 ± 8.9	0.278
T2	22.9 ± 0.3	38.3 ± 1.6	0.317
T3	31.1 ± 0.5	35.5 ± 1.7	0.227

### Posture and behaviour

The percentage of time spent in different postures varied significantly depending on the thermal treatment applied (*p* < 0.001), but it did not vary according to the time period (i.e., the first 4 h vs. hours 4–8), nor was there any interaction between thermal treatment and time period. Therefore, the results by thermal treatment are shown in Table 3.

**Table 3:** Percentage of time spent in the different postures adopted by rabbits according to thermal treatment over an 8-h period.

Posture, %*	Thermal treatment			SE	*p*-value
	T1	T2	T3		
Lying	63.4a	40.9b	5.5c	2.8	<0.001
Sitting	27.5a	19.9b	6.8c	1.2	<0.001
Lateral sprawled	2.7c	15.7b	50.0a	3.9	<0.001
Sternal sprawled	5.7c	22.3b	36.6a	3.3	<0.001
Upright	0.7	1.2	1.1	1.3	0.362

*Percentage based on frame analysis every 5 min (96 frames per rabbit; six rabbits per thermal treatment; 576 frames per thermal treatment). T1: 15.3°C and 63.0% RH; T2: 22.9°C and 38.3% RH; and T3: 31.1°C and 35.5% RH. SE: standard error. Different subscripts in the same row indicate that the means are statistically different between thermal treatments (*p* < 0.05).

Rabbits exposed to T1 spent most of their time lying down (63.4%) or sitting (27.5%), and only marginally in sternal sprawl (5.7%), lateral sprawl (2.7%), or upright (2.3%) postures. However, in T2, time spent lying down was reduced by 35.5% compared to T1 (T1: 63.4%, T2: 40.9%; *p* < 0.001), and in T3 the reduction was even greater, reaching 91.3% (T1: 63.4%, T3: 5.5%; *p* < 0.001). Additionally, T3 showed an 86.6% reduction in lying behaviour compared to T2 (T2: 40.9%, T3: 5.5%; *p* < 0.001).

The percentage of time the rabbits spent sitting also decreased as the ambient temperature increased (*p* < 0.001). In group T2, the time spent sitting was reduced by 27.6% compared to T1 (T1: 27.5%, T2: 19.9%; *p* = 0.001), while in group T3 the reduction was even more pronounced, reaching 75.3% (T1: 27.5%, T3: 6.8%; *p* < 0.001). Additionally, group T3 showed a 65.8% reduction in sitting time compared to T2 (T2: 19.9%, T3: 6.8%; *p* < 0.001).

On the other hand, the percentage of time spent in lateral or sternal sprawled postures increased with rising ambient temperature (*p* < 0.001). In T2, the time spent in these postures roughly doubled compared to T1 (×5.8 and ×3.9, lateral or sternal respectively; *p* < 0.001), and in group T3 the increase was even greater (×18.5 and ×6.9, lateral or sternal respectively; *p* < 0.001). Moreover, group T3 showed an increase of ×3.2 and ×1.8 compared to T2 in time spent sprawled lateral or sternal, respectively (both *p* < 0.001).

There were no significant differences in the time rabbits spent standing upright among thermal treatments (T1: 0.7%, T2: 1.2%, T3: 1.1%; *p* > 0.05).

**Figure 2:**
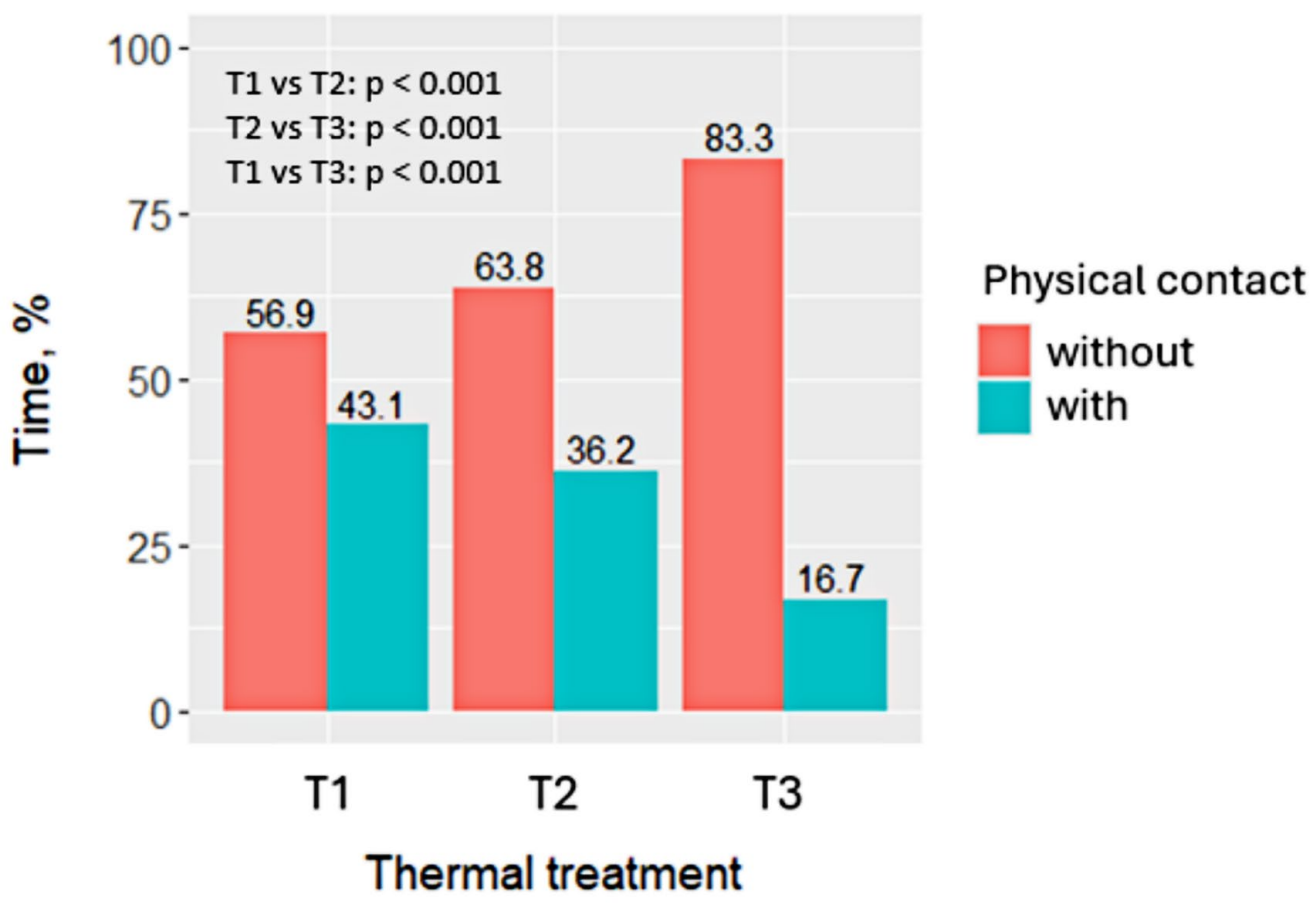
Percentage of events in which rabbits were without or with physical contact in the cage according to the thermal treatment applied. Behaviour was recorded over an 8-h period, and analysed from video frames taken every 5 min (96 frames per rabbit; six rabbits per treatment; 576 frames per thermal treatment). Treatments were: T1 (15.3°C and 63.0% RH), T2 (22.9°C and 38.3% RH), and T3 (31.1°C and 35.5% RH).

The percentage of time rabbits spent in physical contact with another rabbit also varied significantly depending on the thermal treatment applied (*p* < 0.001), but it did not vary according to the time period (i.e., the first 4 h vs. 4–8 h), nor was there any interaction between thermal treatment and time period. Therefore, the results by thermal treatment are shown in Figure 2.

The time rabbits spent in contact with another rabbit was 43.1% in T1. In T2, rabbits spent 36.2% of the time seeking contact with another rabbit, but this did not differ statistically from T1 (*p* = 0.534). However, rabbits in T3 significantly reduced the time spent in contact with the other rabbit compared to T1 (−16.7%; *p* < 0.001). Additionally, group T3 showed a 51.4% reduction in the time spent apart from their companion compared to T2 (−53.9%; *p* = 0.011).

The percentage of time spent on the different behaviours exhibited by the rabbits according to the thermal treatment applied is shown in Table 4.

**Table 4:** Time spent on each behaviour in rabbits according to thermal treatment during an 8-h period.

Behaviour, %*	Thermal treatment			SE	*p*-value
	T1	T2	T3		
Inactivity,	71.2c	78.4b	92.4a	1.6	<0.001
Self-grooming	17.9a	10.8b	1.8c	1.7	<0.001
Exploring	8.4a	8.9a	4.2b	0.8	<0.001
Walking	2.2	2.1	2.3	0.4	0.961
Allogrooming	1.3	1.0	1.0	0.4	0.615
Hopping	1.0	0.0	0.0	–	–
Social negative	0.0	0.0	0.0	–	–

*Percentage based on frame analysis every 5 min (96 frames per rabbit; six rabbits per thermal treatment; 576 frames per thermal treatment). Thermal treatments were T1 (15.3°C, 63% RH), T2 (22.9°C, 38.3% RH), and T3 (31.1°C, 35.5% RH). SE: standard error. Different letters in the same row indicate that the means are statistically different between thermal treatments (*p* < 0.05).

The T1 rabbits spent most of their time inactive (71.2%), followed by self-grooming (17.9%), exploring (8.4%), and marginally walking (2.2%), allogrooming (1.3%) and hopping (1.0%).

Heat-stress treatments (T2 and T3) had a significant effect on the behaviours of self-grooming, exploring, and inactivity (*p* < 0.001). Thus, T2 rabbits reduced self-grooming by 39.7% compared to T1 (T1: 17.9% vs. T2: 10.8%; *p* < 0.001), and in the T3 group, the decrease was even greater, reaching a reduction of 89.9% (T1: 17.9% vs. T3: 1.8%; *p* < 0.001). Additionally, T3 showed an 83.1% reduction in the time spent self-grooming compared to T2 (T3: 1.8% vs. T2: 10.8%; *p* < 0.001).

The time rabbits spent exploring also decreased proportionally with the increase in ambient temperature (*p* < 0.001). There were no differences in the duration of exploration between T1 and T2 rabbits (T1: 8.4%, T2: 8.9%; *p* = 0.920). However, there was a 50% reduction in the duration of exploration in T3 compared to T1 (T1: 8.4%, T3: 4.2%; *p* = 0.002) and a 52.8% reduction between T3 and T2 (T2: 8.9%, T3: 4.2%; *p* < 0.001).

The duration of inactivity also increased significantly with rising ambient temperature (*p* < 0.001). Thus, T2 rabbits increased the duration of inactivity by 10.1% compared to T1 (T1: 71.2%, T2: 78.4%; *p* = 0.007), and in the T3 group, the increase was 29.8% compared to T1 (T1: 71.2%, T3: 92.4%; *p* < 0.001). Furthermore, T3 showed an increase in the duration of inactivity time compared to T2 (+17.9%; *p* < 0.001).

### Space and height occupied

To estimate the minimum space allowance, it was observed that rabbits with a body weight of 2.2–2.3 kg occupied an area equivalent to 168 cm^2^/kg when lying down. In the sprawled positions, sternal and lateral, the space occupied increased to 205 cm^2^/kg and 277 cm^2^/kg, respectively (Table 5).

**Table 5:** Mean (±SD) space occupied per rabbit, and the equivalent available space and stocking density according to the posture adopted by fattening rabbits.

Posture	Space occupied, cm^2^	Available space, cm^2^/kg	Stocking density, kg/m^2^
Lying	382 ± 38	168 ± 17	60 ± 0.1
Sternal sprawled	466 ± 18	205 ± 8	49 ± 0.1
Lateral sprawled	630 ± 82	277 ± 36	36 ± 0.1

To ensure that all rabbits can lie down at the same time, the stocking density should not exceed 60 kg/m^2^. If the rabbits need to stretch out in sternal recumbency, the limit should be reduced to 49 kg/m^2^, and if they need to do so in lateral recumbency, to 36 kg/m^2^.

The range of height occupied by the rabbits was from a minimum of 13 cm when in a sprawled sternal position to a maximum of 32 cm when standing with their head and ears upright.

## Discussion

Posture and behaviour results indicate that the combination of temperature and relative humidity were sufficient to simulate three different thermal conditions.

The objective of this study was to evaluate the posture and behaviour of rabbits of commercial body weight according to ambient temperature and humidity. This aimed to determine the minimum space allowance and height needed to allow rabbits to perform their behavioural needs, especially for thermoregulation purposes.

Regarding posture, under T3 conditions, the most frequent positions and behaviour were lateral and sternal sprawled. These can be interpreted as a heat adaptation mechanism to maximize surface contact with the floor and dissipate heat via conduction. Similar findings have been reported in other studies, indicating that a sprawled position in lateral or sternal recumbency is a reliable indicator of heat stress (9). This posture facilitates heat dissipation through the cage grid by conduction while reducing physical activity by resting, thus preventing increases in body temperature (10). Consequently, the recommended stocking density for rabbit transport should range between 36 and 60 kg/m^2^ depending on thermal conditions, allowing rabbits to adopt their preferred resting postures under each thermal scenario (36 kg/m^2^ to rest in a lateral sprawled position and 60 kg/m^2^ to rest in a lying position).

It is worth noting that the area occupied by rabbits when lying down, as determined in this study, differs from estimates obtained using allometric equations developed by Baxter (11), cited by Petherick and Phillips (4) and referenced in EFSA (1). For instance, in our study, rabbits of 2.27 kg body weight occupied an average space of 382 cm^2^ while lying down, compared to the 468 cm^2^ estimated by the equation, which represents a deviation of 18%. Petherick and Phillips (4) acknowledge that their formula was originally designed for birds, pigs, and cattle, assuming extrapolation to other mammals, but no prior studies have empirically validated its applicability to rabbits.

On the other hand, Giersberg et al. (5) measured the space occupied by Grimoud Freres rabbits using the “KobaPlan” software (12), cited in Giersberg et al. (5). This software calculates the ground area from top-down images, using scale references and adjusting for posture and perspective. The area was computed by digital pixel counting and a linear prediction equation based on rabbit live weight was developed. In their study, the average surface for rabbits in the lying position (“sitting” as per their terminology) was 416 cm^2^, 9% higher than our 382 cm^2^. For sprawled sternal posture, they estimated 535 cm^2^, 15% more than our 466 cm^2^. However, for sprawled lateral posture, their equation estimated 565 cm^2^, while our measurements averaged 630 cm^2^, an 11.5% increase compared to their estimate.

Regarding rabbit behaviour, thermal treatment significantly affected the duration spent on self-grooming, exploration, inactivity, and tendencies to seek physical contact with conspecifics. As ambient temperature increased, time spent self-grooming and exploring decreased while inactivity increased, a pattern consistent with previous research (9). This aligns with findings that rabbits reduce physical activity under heat stress to avoid rises in body temperature (9). Since self-grooming and exploration require greater metabolic activity, these behaviours tend to be suppressed at higher temperatures to conserve energy and facilitate thermal regulation. Furthermore, reductions in these behaviours may reflect a negative impact on overall welfare, as they are associated with fulfilling animals’ natural needs (13).

Additionally, under T3 conditions, rabbits tended to avoid contact with their cage mates compared to T1 and T2, likely as an attempt to dissipate heat and avoid thermal load from close contact. This behaviour reflects a physiological response, as proximity increases body heat exchange between individuals. Previous studies have observed that many species, including rabbits, modify social interactions during thermal stress by seeking greater distances to reduce additional heat exposure from conspecifics (14).

In the context of rabbit transport in the EU, the most common stocking densities range between 60 and 90 kg/m^2^, commonly around 70 kg/m^2^ (European Rabbit Association, personal communication). Therefore, the maximum recommended density of 60 kg/m^2^ under T1 conditions proposed here is slightly lower than that typically used commercially. It is important to highlight that as heat stress increased, rabbits tended to distance themselves from their conspecifics most of the time, suggesting that the minimum available space of 177–205 cm^2^/kg (i.e., stocking density of 56.5–48.8 kg/m^2^) may be insufficient. Greater space would not only allow rabbits to lie in preferred sprawled lateral or sternal positions but also ensure greater separation, which could help reduce heat stress and improve welfare.

Regarding height, rabbits in sternal or lateral sprawled positions, even under heat stress conditions (T2 or T3), did not raise their ears to dissipate heat, occupying a height equal to or less than 13 cm. However, in all thermal conditions tested, rabbits occasionally stood upright, reaching heights up to 32 cm. Therefore, a minimum cage height of 35 cm would allow rabbits to change posture and stand without their heads touching the cage grid.

It is important to emphasize that this study was conducted under experimental conditions using cages of 35 cm height as transport containers with an available space of 714 cm^2^/kg (i.e., stocking density of 14 kg/m^2^), housing two rabbits per cage. Thus, the effects of varying thermal conditions (temperature, relative humidity, wind speed), available space, and container height in commercial transport on behaviour, thermophysiological, and metabolic responses remain unknown. Consequently, this study serves only as a preliminary approach to defining space and container height treatments for evaluation in future studies.

As a next step, a follow-up study has already been carried out under controlled experimental conditions to specifically assess the combined effects of different space allowances and container heights under a range of thermal scenarios. The final validation of science-based recommendations for rabbit transport will rely on the integration of results from the present study, the follow-up experimental trial, and a third study currently underway under real commercial transport conditions. Together, these studies aim to provide robust empirical evidence to inform future regulatory decisions and support the development of species-specific requirements for rabbits in the revision of Council Regulation (EC) No 1/2005, in line with the objectives of the Farm to Fork Strategy.

## Conclusion

A minimum available space of 168 cm^2^/kg, or the equivalent of a maximum stocking density of 60 kg/m^2^ under thermal comfort conditions, is established to allow all animals to lie down simultaneously. For all rabbits to sprawl laterally or sternally to dissipate heat, under static conditions without ventilation, an available space of 205 cm^2^/kg is required for sternal sprawl (equivalent to a maximum stocking density of 36 kg/m^2^), and 277 cm^2^/kg (equivalent to a maximum stocking density of 49 kg/m^2^). Increasing the available space beyond these minimums may further facilitate heat loss and help prevent full-body contact between animals, thus improving thermal comfort. The minimum cage height that allows rabbits of commercial weight (2.0–2.5 kg live weight) to change posture and stand upright without their heads touching the cage grid is set at 35 cm.

Rabbit posture and behaviour did not differ significantly between the 4-h and 8-h time points. The most common behaviour observed was inactivity, followed by self-grooming, regardless thermal conditions. However, as thermal conditions became more challenging, self-grooming decreased while inactivity increased further. Rabbits remained in an upright position approximately 1% of the time, irrespective of the thermal conditions.

Given the methodological limitations, these recommendations should be explicitly framed as preliminary indications that require validation under commercial transport conditions.

## Data Availability

The raw data supporting the conclusions of this article will be made available by the authors, without undue reservation.
